# Contactin proteins in cerebrospinal fluid show different alterations in dementias

**DOI:** 10.1007/s00415-024-12694-6

**Published:** 2024-09-25

**Authors:** Besnik Muqaku, Sarah Anderl-Straub, Leonie Werner, Magdalena Nagl, Markus Otto, Charlotte E. Teunissen, Patrick Oeckl

**Affiliations:** 1 German Center for Neurodegenerative Diseases (DZNE E.V.), Helmholtzstr. 8/1, 89081 Ulm, Germany; 2 Department of Neurology, Ulm University Hospital, Helmholtzstr. 8/1, 89081 Ulm, Germany; 3 Department of Neurology, Martin-Luther-University Halle-Wittenberg, Halle (Saale), Germany; 4 Neurochemistry Laboratory, Department of Clinical Chemistry, Amsterdam University Medical Centers (UMC), Amsterdam, The Netherlands

**Keywords:** Contactin, Biomarker, Cerebrospinal fluid, Synaptic dysfunction, Dementia, Alzheimer’s disease

## Abstract

**Background:**

The proteins contactin (CNTN) 1–6 are synaptic proteins for which there is evidence that they are dysregulated in neurodegenerative dementias. Less is known about CNTN changes and differences in cerebrospinal fluid (CSF) of dementias, which can provide important information about alterations of the CNTN network and be of value for differential diagnosis.

**Methods:**

We developed a mass spectrometry-based multiple reaction monitoring (MRM) method to simultaneously determine all six CNTNs in CSF samples using stable isotope-labeled standard peptides. The analytical performance of the method was evaluated for peptide stability, dilution linearity and precision. CNTNs were measured in 82 CSF samples from patients with Alzheimer’s disease (AD, *n* = 19), behavioural variant frontotemporal dementia (bvFTD, *n* = 18), Parkinson’s disease dementia/dementia with Lewy bodies (PDD/DLB, *n* = 18) and non-neurodegenerative controls (*n* = 27) and compared with core AD biomarkers.

**Results:**

The MRM analysis revealed down-regulation of CNTN2 (fold change (FC) = 0.77), CNTN4 (FC = 0.75) and CNTN5 (FC = 0.67) in bvFTD and CNTN3 (FC = 0.72), CNTN4 (FC = 0.75) and CNTN5 (FC = 0.73) in PDD/DLB compared to AD. CNTN levels strongly correlated with each other in controls (*r* = 0.73), bvFTD (*r* = 0.86) and PDD/DLB (*r* = 0.70), but the correlation was significantly lower in AD (*r* = 0.41). CNTNs in AD did not show correlation even with core AD biomarkers. Combined use of CNTN1-6 levels increased diagnostic performance of AD core biomarkers.

**Conclusions:**

Our data show CNTNs differentially altered in dementias and indicate CNTN homeostasis being selectively dysregulated in AD. The combined use of CNTNs with AD core biomarkers might help to improve differential diagnosis.

**Supplementary Information:**

The online version contains supplementary material available at 10.1007/s00415-024-12694-6.

## Introduction

Synapses are the functional connections primarily between neurons and are essential for memory formation. Conversely, synaptic dysfunction or degeneration is strongly associated with memory impairment [1], and a common feature across various forms of dementia such as Alzheimer’s disease (AD).

Synaptic loss is an early event in Alzheimer’s disease (AD) pathogenesis [2, 3] and shows stronger correlation with cognitive dysfunction than amyloid and tau pathology [4, 5]. Synaptic markers are therefore a highly desired need for the diagnostic work-up of patients, prognosis and to evaluate treatment effects. Fluid biomarkers of synaptic dysfunction also offer the opportunity to study synaptic alterations in neurodegenerative dementias and differences between the diseases.

Several synaptic biomarker candidates in cerebrospinal fluid (CSF) and blood have already been studied in dementias such as *β*-synuclein [6, 7], neurogranin [8], SNAP-25 [9], and GAP-43 [10] which reflect different processes and synaptic compartments. Many synaptic fluid biomarkers are increased in AD [8–16] but are not regulated in other dementia patients, such as behavioral variant frontotemporal dementia (bvFTD) and Parkinson’s disease dementia/dementia with Lewy bodies (PDD/DLB), or show decreased levels compared to controls and AD [10–13, 15, 16]. This discrepancy strongly supports spatial and temporal differences of synaptic degeneration [11, 13] or different synaptic pathology between dementias [11] and highlights the need for a more detailed characterization and comparison of synaptic dysfunction between these diseases.

Contactins (CNTNs), including the proteins CNTN1 to CNTN6 (CNTN1-6), belong to the Immunoglobulin (Ig) superfamily and are structurally strongly related [17, 18]. They are predominantly expressed in the brain without specific localization to a certain brain area [19, 20]. Although CNTNs are mainly expressed in neurons, they were also found in oligodendrocytes and their precursor cells [21, 22]. In neurons, CNTNs are located in axons and synapses and are involved in the establishment of synaptic contacts, synaptic receptor function, and dendritic spine morphology [19]. Thereby, they play a pivotal role in many neuron-related processes, such as the organization of axonal domains, axonal guidance, myelination, neuritogenesis, neuronal development, synaptogenesis, axo-glia interactions, and neural circuit [19]. There is evidence linking CNTNs and their interaction partners with neurodegeneration [18, 19, 23]. For instance, CNTNs interact with the amyloid precursor protein (APP) and knock-out of several of the CNTN family members lead to synaptic and memory dysfunction [18, 19].

So far and in separate studies, changes of only CNTN1, CNTN2, and CNTN5 CSF levels have been reported in neurodegenerative dementias and the results for CNTN2 being altered in AD were not always congruent [24–27]. However, the assessment of the level of all CNTNs in CSF can provide important information about alterations of the CNTN network in neurodegenerative diseases and may uncover their potentially distinct role in dementias.

The aim of the present study was to develop a mass spectrometry-based method for the simultaneous determination of all six CNTNs (CNTN1-6) in CSF using multiple reaction monitoring (MRM). CSF levels of CNTN1-6 were measured with the developed methods in a pilot cohort of patients to investigate disease-related changes and differences between dementias including AD (*n* = 19), bvFTD (*n* = 18) and DLB/PDD (*n* = 18).

## Methods

### Patients

CSF samples were collected during diagnostic work-up of patients at the Ulm University Hospital, Department of Neurology and included patients diagnosed with AD, bvFTD, DLB, PDD and non-neurodegenerative controls where CSF was collected to rule out a neuroinflammatory condition. Diagnoses among control patients included 11 × facial palsy, 6 × tension headache, 2 × trochlear nerve palsy, 1 × intoxication, 1 × migraine, 1 × ocular myositis, 1 × pain syndrome right leg, 1 × pansinusitis, 1 × physical and mental stress and prostate cancer, 1 × polyneuropathy and restless leg syndrome, 1 × vertigo. Diseases were diagnosed according to established criteria [28–31]. CSF was collected by lumbar puncture, centrifuged and stored within 2 h at − 80 °C in polypropylene tubes. CSF Tau, pTau181 and Aβ42 were measured by ELISAs from Fujirebio Germany GmbH (Hannover, Germany) during routine clinical assessment.

### CSF sample preparation for MRM analysis

For in-solution digestion, a solution containing TEAB (triethylammonium bicarbonate), TCEP (tris(2-carboxyethyl) phosphine hydrochloride), CAA (2-chloroacetamide), and stable isotope-labeled standard peptides (Table S1) was added to 200 µL CSF sample, giving a final concentration of 100 mM TEAB, 5 mM TCEP and 10 mM CAA. Proteins were reduced and alkylated by incubating the sample for 10 min at 95 °C and 400 rpm. Protein digestion took place overnight at 37 °C after adding Trypsin/LysC (Promega) at an enzyme-to-protein ratio of 1:50. Digestion was stopped with 1% trifluoroacetic acid (TFA) final concentration. Digested peptides were fractionated with strong cation exchange (SCX) STAGE Tips (Affinisep SPE-Disks-Bio-SCX-47.20). Peptide fractionation was performed using different concentrations of ammonium acetate (75, 125, 200, 300, 450 mM) in 20% acetonitrile (ACN) and 0. 5% formic acid (FA), while fraction six contained 80% ACN and 5% ammonium hydroxide. After vacuum drying, samples were reconstituted in 27.5 µL 6% ACN and 0.1% TFA.

### MRM analysis

For MRM analysis, 20 µL of the fractionated sample was injected onto a C18 PepMap100, 5 μm, 0.3 × 5.0 mm trap column (Thermo) using an Agilent 1260 HPLC system operating at a flow rate of 200 µL/min. Solvent composition: A—0.05% TFA in water and B—0.05% TFA in methanol. An Eksigent MicroLC200 chromatographic system was used to separate peptides with an Eksigent HALO Fused-core C18, 2.7 μm, 0.5 × 100 mm analytical column with a gradient time of 10 min and total run time of 15.5 min (flow rate of 15 µL/min). Solvent composition used for peptide separation: A—4% DMSO, 0.1% FA and B—96% ACN, 4% DMSO, 0.1% FA. Ionized peptides were analyzed on a QTRAP 6500 mass spectrometer in positive ion mode (AB Sciex, Darmstadt, Germany). MRM settings are described in Table S1. Skyline software was used for the evaluation of all MRM data [32], and the data were reported as abundance ratio of endogenous peptides and their respective labeled standard peptides (light/heavy (L/H) ratio).

### MRM method development and validation

For each CNTN at least two most abundant peptides from proteomics screening experiments with CSF samples [33] were included in the panel of peptides for MRM method development. Isotopic labeled standard peptides, QPrESTs (Atlas Antibodies AB, Bromma, Sweden) or AQUA peptides (Thermo Fisher Scientific), were custom-synthesized for all peptide candidates and used for MRM method development (Table S1) [33]. The final MRM method included the best three transitions per peptide. For CNTN5, only one transition was used for quantification because the others showed interferences in patient samples. Finally, the MRM consisted of 18 peptides (endogenous and labeled standard peptides) derived from CNTN1-6. A CSF pool sample was used to evaluate the analytical performance of the developed MRM method. Here, the stability of endogenous peptide was tested after several times of freezing and thawing of the sample. The dilution linearity of the peptides was assessed by diluting the sample up to eightfold with artificial CSF (aCSF, EcoCyte), and intra-assay variation was investigated by replicate measurement of the CSF pool sample (*n* = 5).

### Statistical analysis

Statistical analyses were performed using GraphPad Prism v.6 and R software v. 4.1.0. Groups were compared by Kruskal–Wallis test corrected with Dunn’s post hoc test for multiple comparisons. Correlation analyses were performed using Spearman’s rank correlation coefficient. Receiver operating characteristic (ROC) curves were generated in R v. 4.1.0 by using the package pROC. For the multivariate ROC curves, the multinomial logistic regression was implemented using nnet R package and the multinomial log-linear model. A *p*-value < 0.05 was regarded statistically significant.

## Results

### MRM method development and validation

CNTN peptide candidates for MRM method development were selected based on CSF proteomics screening data [33]. Peptides containing methionine residue were excluded and at least two most abundant peptides per CNTN were chosen. We used stable isotope-labeled standard peptides for method development and thereby selected the best fragments and optimized collision energy, entrance potential, collision cell exit potential and prefilter potential. In the end, only peptides for which endogenous counterparts showed good and interference-free signal in CSF pool sample were considered for further evaluation (Table S1). Except CNTN4 which is represented by only the canonical protein variant, all conformed protein isoforms of other CNTNs contain the sequence of measured peptides (Table S1). Assay performance for all CNTN peptides was validated using a CSF pool sample regarding dilution linearity, stability and precision (Table 1). All peptides were stable for 2 h at room temperature and up to five freeze–thaw cycles. All peptides showed dilution linearity up to eightfold dilution with the exception of CNTN3 and CNTN5. CNTN5 showed the lowest abundance among all CNTNs and dilution linearity was shown for twofold dilution. Higher dilutions were below the detection limit and could not be tested. The CNTN3 peptide showed > 20% deviation for the 1:4 and 1:8 dilution indicating some interferences that need to be considered during data interpretation. Assessment of intra-assay variations revealed a coefficient of variation (CV) < 5% for all peptides, except for CNTN5 with a CV of 11.6%.

**Table 1:** MRM assay performance

Protein gene name	Stability test (*n* = 2)				Dilution linearity (*n* = 2)			Intra-assay variation (*n* = 5)
	2 h RT	1 cycle	3 cycles	5 cycles	1 to 2	1 to 4	1 to 8	
CNTN1	98.6–99.7	96.0–96.5	92.4–98.3	93.5–97.1	88.8–103.6	81.8–88.3	85.9–90.6	1.3
CNTN2	100.5–100.8	96.4–101.0	97.0–101.4	100.5–103.9	98.5–105.8	101.8–102.3	101.9–104.5	0.5
CNTN3	94.6–95.6	99.7	88.1–94.0	93.1–95.7	114.2–116.5	138.1–138.8	146.1–148.7	3.4
CNTN4	95.5–100.0	93.9–100.3	95.5–103.0	90.3–102.2	109.3–110.9	106.3–109.6	117.1–129.0	1.6
CNTN5	92.3–97.2	119.7–126.2	102.1–97.4	95.3–106.7	115.5–100.4			11.6
CNTN6	98.2–106.3	99.1–104.3	97.3–99.6	108.0–102.5	99.5–113.1	103.9–107.1	109.2–109.6	5

2 h RT—incubated for 2 h at room temperature; 1 cycle—one freeze–thaw cycle; 3 cycles—three freeze–thaw cycles; 5 cycles—five freeze–thaw cycles. *n*—number of replicates

### Different abundance of CNTNs in neurodegenerative dementias

The validated MRM method was applied to measure CNTNs in 82 CSF samples including control patients without neurodegenerative diseases and sex- and age-matched patients with neurodegenerative dementias (Table 2). The cohort comprised 27 controls (Con), 19 AD, 18 bvFTD and 18 PDD/DLB patients. The variation in quality control samples (QC) measured in the same batch with patient samples was < 10% for all peptides. Technical issues with chromatography led to missing values for some samples and peptides. Values were missing for: CNTN2—2 patients (1 AD and 1 PDD/DLB), CNTN3—6 patients (1 Con, 1 AD, 2 bvFTD, 2 PDD/DLB), CNTN4—7 patients (2 Con, 2 AD, 1 bvFTD, 2 PDD/DLB, CNTN5—3 patients ( 2 AD and 1 PDD/DLB), and CNTN6—1 PDD/DLB patient.

**Table 2:** Patient characteristics

	Total	Control	AD	bvFTD	PDD/DLB
Patient Nr	82	27	19	18	18
Female (%)	27 (33%)	9 (33%)	7 (37%)	6 (33%)	5 (28%)
Age (years, median) Min.—Max.	69 (46–81.8)	68.7 (48.1–81.7)	73.6 (65–81)	64.2 (46 –69)	73 (62 –81.8)

*AD* Alzheimer's disease, *bvFTD* behavioral variant of frontotemporal dementia, *PDD* Parkinson’s disease dementia, *DLB* dementia with Lewy bodies

**Fig. 1:**
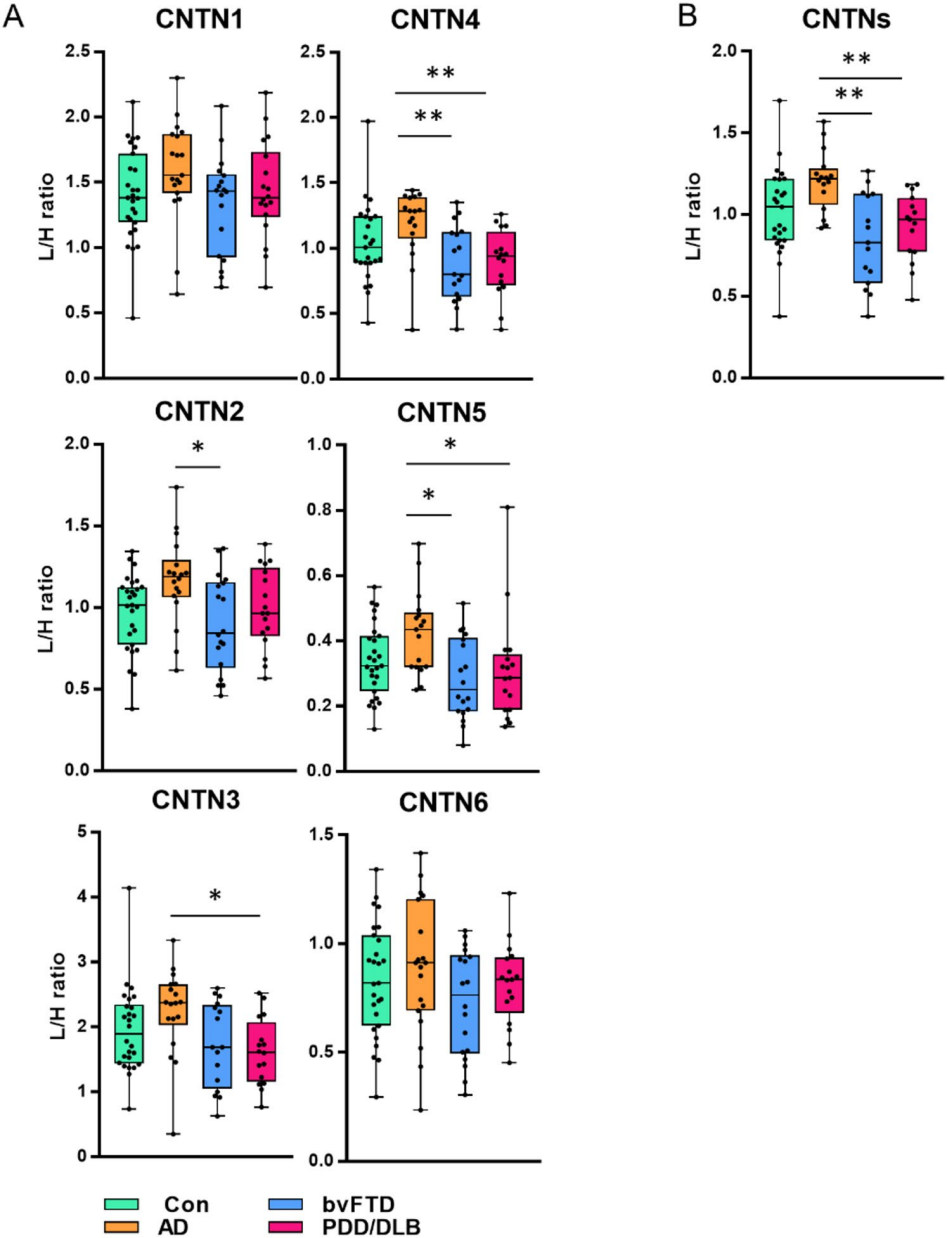
Comparison of CSF CNTN levels between neurodegenerative dementias. Contactins (CNTN) 1–6 were measured in CSF by multiple reaction monitoring (MRM) in patients with Alzheimer’s disease (AD), behavioural variant frontotemporal dementia (bvFTD), dementia with Lewy bodies/Parkinson’s disease dementia (DLB/PDD) and non-neurodegenerative controls (Con). Boxplots show the light-to-heavy peptide ratio (L/H ratio) of MRM data –for **A** CNTN 1–6 and **B** the normalized average of all contactins (CNTNs). Kruskal–Wallis test corrected with Dunn’s factor for multiple comparisons was applied to investigate differences in protein level overall patient groups. *p-value < 0.05; **p-value < 0.01

We observed a slight abundance increase of all CNTNs in AD compared to controls, even though this was not significant for any of the CNTNs (Fig. 1A). Several CNTNs were significantly regulated between dementia patients. Compared to AD patients, we observed down-regulation of CNTN2 (fold change (FC) = 0.77, CNTN4 (FC = 0.75) and CNTN5 (FC = 0.67) in bvFTD, and CNTN3 (FC = 0.72), CNTN4 (FC = 0.75) and CNTN5 (FC = 0.73) in PDD/DLB. Although not all CNTNs are significantly regulated, their abundance shows similar trends across all patient groups. Next, we combined all CNTNs in a single score by averaging their normalized ratio. The ratio for each CNTN was normalized to the average ratio value of respective CNTN over all samples. The combined score showed a significant decreased in bvFTD (FC = 0.70) and PDD/DLB (FC = 0.76) compared to AD (Fig. 1B).

### Strong correlation between CNTNs in bvFTD but not in AD patients

**Fig. 2:**
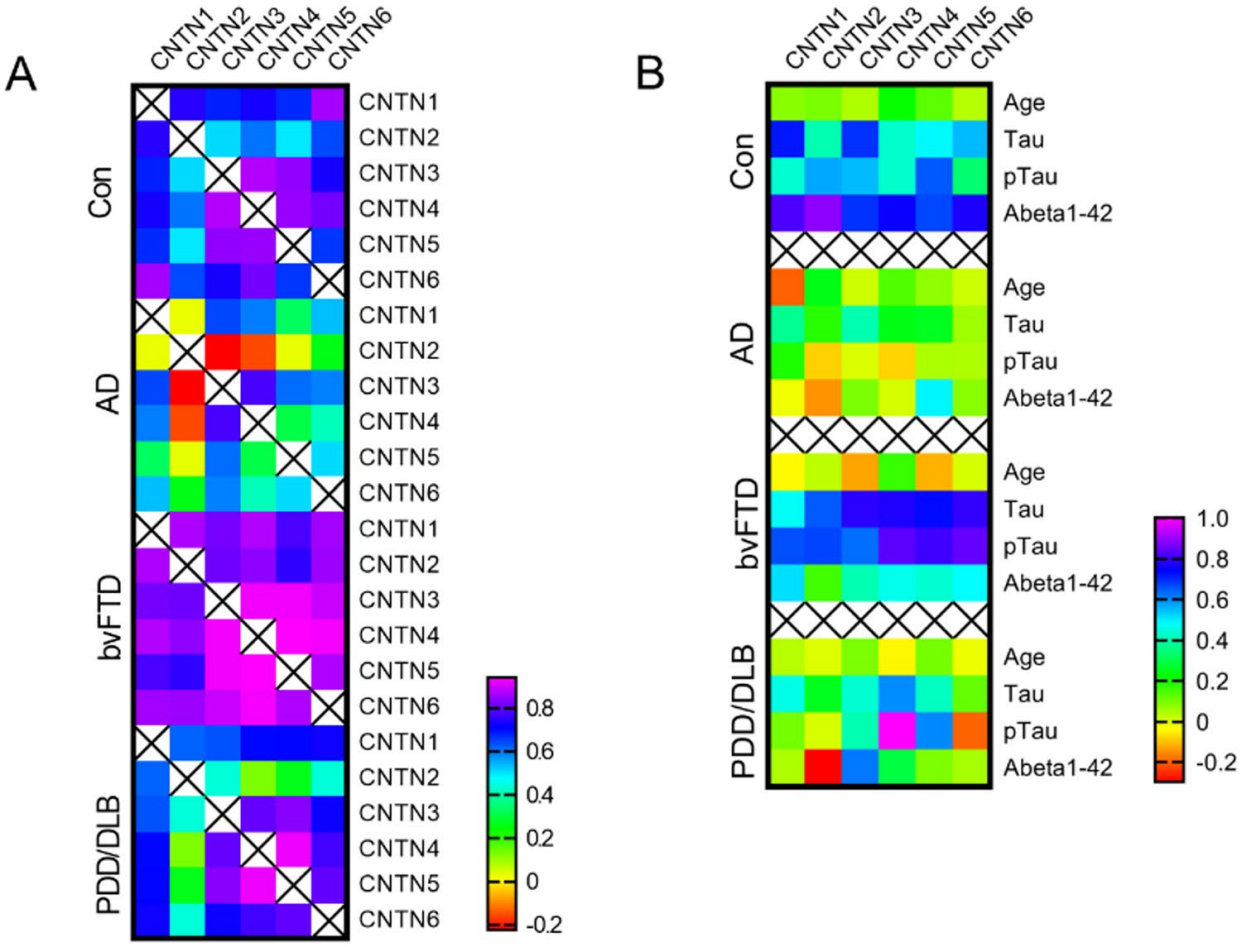
Correlation of CNTNs with age and other biomarkers. Heat map representing the Spearman’s rank correlation coefficient within each patient group for the correlation of CSF levels of contactins (CNTN) 1–6 with **A** each other and **B** with age and other biomarkers. *AD* Alzheimer’s disease, *bvFTD* behavioural variant frontotemporal dementia, *DLB/PDD* dementia with Lewy bodies/Parkinson’s disease dementia, *Con* non-neurodegenerative controls

We next investigated the association of the CNTNs with each other in the four patient groups. The levels between CNTNs correlated strongly in control samples with a median spearman’s correlation coefficient (*r*) of 0.73 (Fig. 2A). We observed a similar strong correlation of CNTNs in bvFTD (*r* = 0.86) and PDD/DLB samples (*r* = 0.70) but it was substantially lower within AD samples (*r* = 0.41). Also, the correlation between all CNTNs with established biomarkers was dramatically lower for AD and PDD/DLB compared to controls (*r*; Tau (AD = 0.24, PDD/DLB = 0.42, Con = 0.51), pTau (AD = 0.02, PDD/DLB = 0.25, Con = 0.49) and Aβ42 (AD = 0.04, PDD/DLB = 0.07, Con = 0.76)) (Fig. 2B). In contrast, Tau, pTau show a better correlation with CNTNs in bvFTD than controls (*r* in bvFTD; Tau = 0.75, pTau = 0.74), and only the correlation of CNTN1 with Tau became lower in bvFTD. However, the correlation of CNTNs with Aβ42 showed lower *r*-values in bvFTD than controls (*r*; bvFTD = 0.45, Con = 0.76). None of the CNTNs correlated with age within all patient groups (*r*; Con = 0.09, bvFTD = − 0.02, AD = 0.04, PDD/DLB = 0.01).

### CNTNs provided an added value to the established CSF biomarkers

**Fig. 3:**
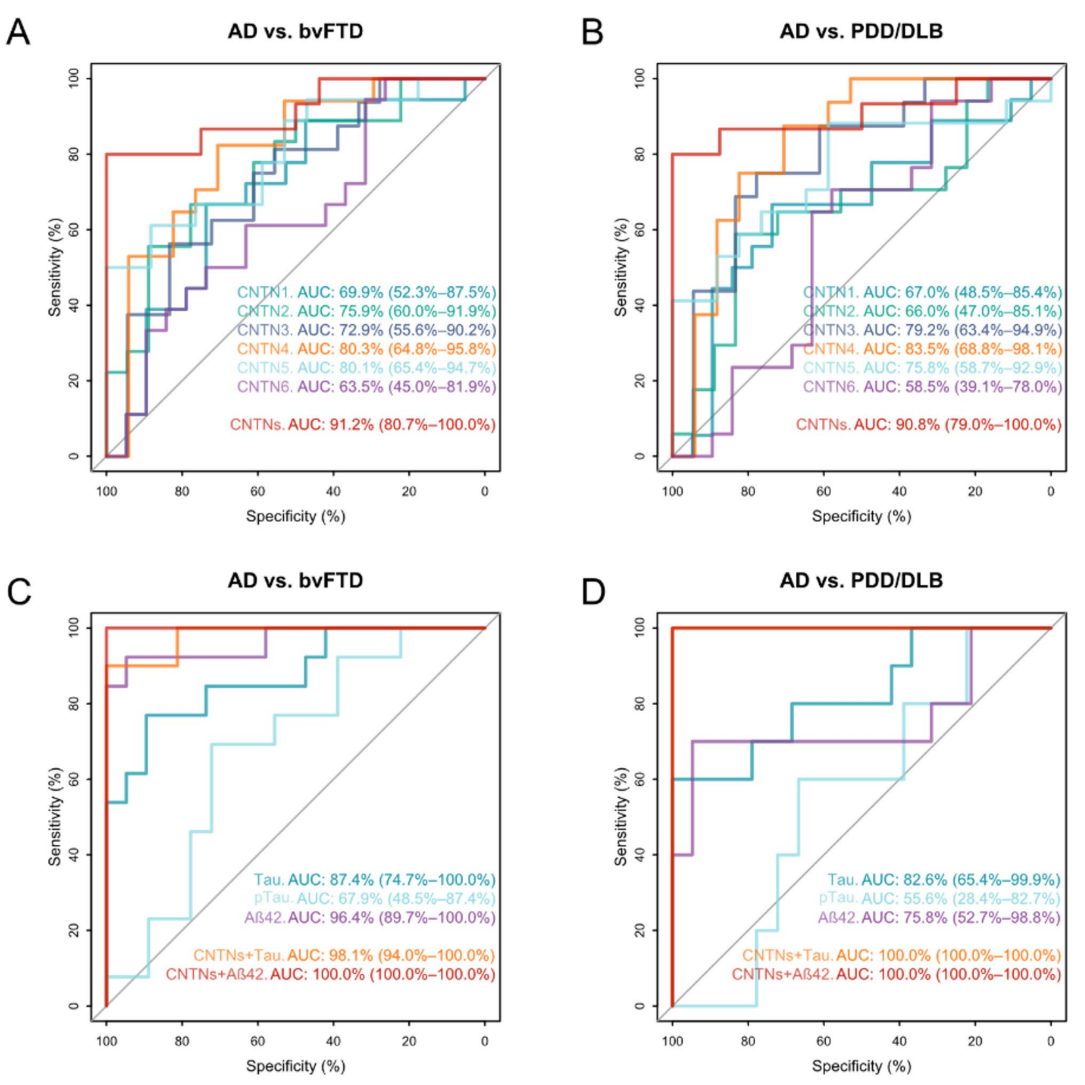
Diagnostic performance of CNTNs. Receiver operating characteristic (ROC) curve analysis of CSF levels of contactins (CNTN) 1–6 (**A**, **B**), core AD biomarkers and biomarker combinations (**C**, **D**). Data are the area under the curve (AUC) and 95% confidence interval. *AD* Alzheimer’s disease, *bvFTD* behavioural variant frontotemporal dementia, *DLB/PDD* dementia with Lewy bodies/Parkinson’s disease dementia, *Con* non-neurodegenerative controls

We performed receiver operating characteristic (ROC) curve analysis to investigate the potential of CNTNs as biomarkers for differentiating AD patients from bvFTD and PDD/DLB (Fig. 3). CNTN4 provided the highest area under the curve (AUC) value from all CNTNs for the differentiation of AD from bvFTD (AUC = 80.3%) and PDD/DLB (AUC = 83.5%). A multivariate ROC curve including all CNTNs revealed an AUC value of 91.2% for AD vs. bvFTD and 90.8% for AD vs. PDD/DLB. From the established CSF AD biomarkers, only Aβ42 showed a higher AUC (96.4%) than all CNTNs for the discrimination of AD from bvFTD patients whereas CSF Tau and pTau181 showed AUCs of 87.4% and 67.9. The best separation of AD from bvFTD and DLB/PDD patients was achieved by combining CNTNs with Aβ42 and Tau with an AUC value from 98.1 to 100% (Fig. 3).

## Discussion

In the present study, we report the MRM assay development and the simultaneous measurement of all six CNTNs in CSF samples from patients with different neurodegenerative dementias. The validated MRM method revealed different abundance changes of CSF CNTNs 1–6 between dementias which was similar for all CNTNs in all patient groups. The correlation of CNTNs to each other was disrupted only in AD.

Here, we show several CNTNs, axonal and synaptic proteins, significantly down-regulated in bvFTD and PDD/DLB compared to AD. However, all CNTNs exhibit similar trends over all patient groups, compared to controls i.e. non-significant abundance increase in AD and an equal or very slight decrease in bvFTD and PDD/DLB. This observation indicates that, despite the fact that among dementia patients different brain regions are affected and CNTN members are localized in overlapping but different brain areas [19], all CNTNs seem to be equally affected. This conclusion is further supported by the normalized average ratio of all CNTNs showing equal trend as individual CNTNs, and by the strong correlation of all CNTNs with each other. Thus, our data suggest differences in pathological processes for explaining the opposite regulation of synaptic markers in dementia patients rather than affected brain regions.

In contrast to controls, CSF levels of the different CNTNs showed only weak correlation to each other in AD. This probably indicates a perturbation of CNTN interaction networks in neurons, thus impacting synapse function integrity. Indeed, the interaction of CNTNs with APP plays a critical role in AD, potentially by influencing three key processes: APP processing, synaptic plasticity, and neuronal integrity [18]. It has been hypothesized that any dysregulation or imbalance in these processes could lead to aberrant APP processing and contribute to the pathogenesis of AD [18]. Thus, our data in CSF provide further support for an imbalance of CNTN homeostasis in AD. Interestingly, the correlation of CNTNs in bvFTD exhibits an opposite trend compared to AD, highlighting its potential utility in differential diagnosis between different types of dementia. This is supported by our ROC curve analysis showing good discriminatory power of CNTNs alone to distinguish AD from bvFTD and PDD/DLB and an added value when combined with core AD biomarkers.

Although CNTN2 showed a significant difference only between AD and bvFTD, elevated abundance levels in AD compared to controls can be easily recognized. This finding aligns with a study that identified a significant increase of CNTN2 in AD through mass spectrometry analysis conducted on a limited number of samples [26]. Another study in two cohorts with a significantly higher number of patients and by using ELISA, observed down-regulation of CNTN2 in CSF samples of AD patients [25]. The reduced CNTN2 levels in AD have been attributed to its protective function by lowering the production of Aβ peptides induced upon CNTN2 binding with APP [25]. Indeed, the interplay of CNTN2-5 with APP proteins has been demonstrated many times [34–39]. Nevertheless, besides possible discrepancies in patient stratification, the contradictory observations might be related to different applied methodologies. Further, an ELISA targeting a single protein variant might yield different outcomes than MS measuring small protein fragments (peptides) generated after the digestion of proteins or protein fragments. An alternative approach involving peptidomics, which analyzes peptides without protein digestion and separates proteins from their larger fragments (peptides), could help elucidate the observed discrepancies regarding CNTN2 [40].

We observed a non-significant increase of CNTN5 levels in AD and significant differences in AD relative to bvFTD and PDD/DLB. In contrast to our findings, recent research measuring CNTN5 in CSF with an antibody-based proximity extension assay reported decreased levels in AD and even in MCI patients compared to cognitively unaffected individuals [27]. However, there is no analytical validation of this assay reported especially regarding specificity for CNTN5 or whether specific CNTN5 protein isoform was measured, thus hampering the interpretation of the discrepancies with our study. Further examinations are required to clarify the conflicting results.

The strength of this study is the simultaneous investigation of all six CNTNs and the use of the highly specific MRM providing a comprehensive overview of the CNTN system in neurodegenerative dementias. The main limitation of this pilot study is the small sample size which originates from the exploratory nature of this project to provide a basis for the initiation of larger studies in the future.

## Conclusion

In conclusion, our data indicate that CNTN homeostasis is dysregulated in neurodegenerative dementias. CSF CNTN levels show different patterns across dementia disorders and they might provide an added value to the AD core biomarkers in differential diagnosis. The assay developed in our study provide a basis to further study CSF CNTNs in larger patient cohorts.

## Supplementary Information

Below is the link to the electronic supplementary material.

Supplementary file1 (XLSX 18 KB)

## Data Availability

The data supporting the findings of this study are available from the corresponding author upon reasonable request.
